# Stereotactic Re-irradiation of Sternal Metastases Using Skin Surface Fiducial Markers and Real-Time Motion Synchronization

**DOI:** 10.7759/cureus.82239

**Published:** 2025-04-14

**Authors:** Nicolas Bachmann, Hannes A Loebner, Alexander Althaus, Hossein Hemmatazad

**Affiliations:** 1 Department of Radiation Oncology, Inselspital, Bern University Hospital, University of Bern, Bern, CHE

**Keywords:** cyberknife, fiducial, metastasis, motion management, motion mitigation, real-time, re-irradiation, sbrt, stereotactic, sternum

## Abstract

Due to respiratory motion, treating sternal metastases with stereotactic body radiotherapy (SBRT) is challenging, often requiring large irradiation volumes to account for target movement. To address this, we implemented a straightforward approach by placing skin fiducial markers near the sternal metastasis, enabling real-time motion synchronization with the CyberKnife® System (Accuray Inc., Sunnyvale, CA). Advances in anti-cancer therapies have significantly extended survival in metastatic patients, increasing their likelihood of requiring re-irradiation and experiencing late toxicities. We present the outcomes of two patients, one with metastatic hepatocellular carcinoma (mHCC) and one with metastatic breast cancer (mBC), who underwent two courses of SBRT for sternal metastases using the CyberKnife® and skin fiducial markers for motion management. Both patients tolerated the treatment well, achieving complete pain relief and durable local control. No late toxicity was observed in the mHCC case, while the mBC patient developed significant left anterior descending artery (LAD) stenosis, which may have been linked to cumulative radiation exposure. Given the known risk of cardiac toxicity associated with radiation therapy, these findings underscore the importance of minimizing cardiac dose to reduce long-term toxicity, particularly in re-irradiation cases.

## Introduction

Bone metastases (BM) are common in advanced cancer, with the vertebral column and pelvis being the most frequently affected sites [1]. While the sternum’s role in supporting the thoracic spine is well documented in trauma patients, there is limited information on its significance in metastatic disease [2]. External beam radiotherapy (EBRT) is the standard treatment for BM, primarily for pain relief and fracture prevention. As cancer survival improves with advancing therapies, stereotactic body radiotherapy (SBRT) has gained interest for its potential to provide better local control and pain relief through high-dose, ablative treatments. With longer survival, the need for re-irradiation over the course of the disease is potentially increased, and patients are more likely to experience radiation-induced late toxicities, which often manifest only years after treatment. Given the sternum's anatomical proximity to the heart, radiation-induced cardiac toxicities are of particular concern, especially in the re-irradiation setting. Extensive data from thoracic cancer studies have shown that an increased mean heart dose, among other factors, is associated with a higher risk of cardiotoxicity [3]. While the ALARA (As Low As Reasonably Achievable) principle applies at our institution with regard to mean heart dose, we also adhere to the SBRT constraints proposed by Timmerman [4]. For example, they recommend that when delivering SBRT near the heart in 5, 10, or 15 fractions, no more than 15 cc of cardiac tissue should receive a dose exceeding 32 Gy, 36 Gy, or 42 Gy, respectively (D15 cc). Given the lack of established dose constraints in the re-irradiation setting, we aim to adhere to these thresholds when summing up the doses from the first and second SBRT, conservatively assuming no biological dose recovery over time.

Treating sternal metastases with SBRT is particularly challenging due to respiratory motion. Traditional free-breathing treatments require larger irradiation volumes to compensate for motion, leading to higher doses to organs at risk, including the heart. Previously, we reported on the use of the CyberKnife® System (Accuray Inc., Sunnyvale, CA) for sternal metastases, utilizing skin surface fiducial markers (FMs) to mitigate respiratory motion through fiducial tracking and real-time motion synchronization [5]. Four FMs were placed in a diamond-shaped configuration on the skin near the sternal metastasis during planning computed tomography (CT) to serve as surrogates for tumor localization. Their positions were marked with small tattoos to ensure consistent placement of the markers for each SBRT session. The underlying principle mirrors that of traditional treatments using invasively implanted gold markers. At the beginning of every treatment fraction, the FMs were visualized using two orthogonal x-ray images and correlated with the patient’s respiratory cycle via the Synchrony® Respiratory Tracking System (Accuray Inc., Sunnyvale, CA) [6]. These x-ray images were acquired at 30-second intervals throughout the treatment to verify the accuracy of the respiratory model and determine whether adjustments were necessary. A minimum of three FMs is required to correct for translational and rotational movements, while the fourth marker acts as a backup in case one cannot be detected. The default threshold for the maximum rigid body error of 1.5 mm was used. A clinical target volume (CTV) to planning target volume (PTV) margin of 3 mm was applied to account for residual uncertainties.

After successful implementation, this technique was standardized at our institution for sternal oligometastases. Here, we present the outcomes of two re-irradiation cases with sternal metastases who underwent two courses of SBRT using skin surface FMs for motion management.

## Case presentation

Case 1

**Figure 1:**
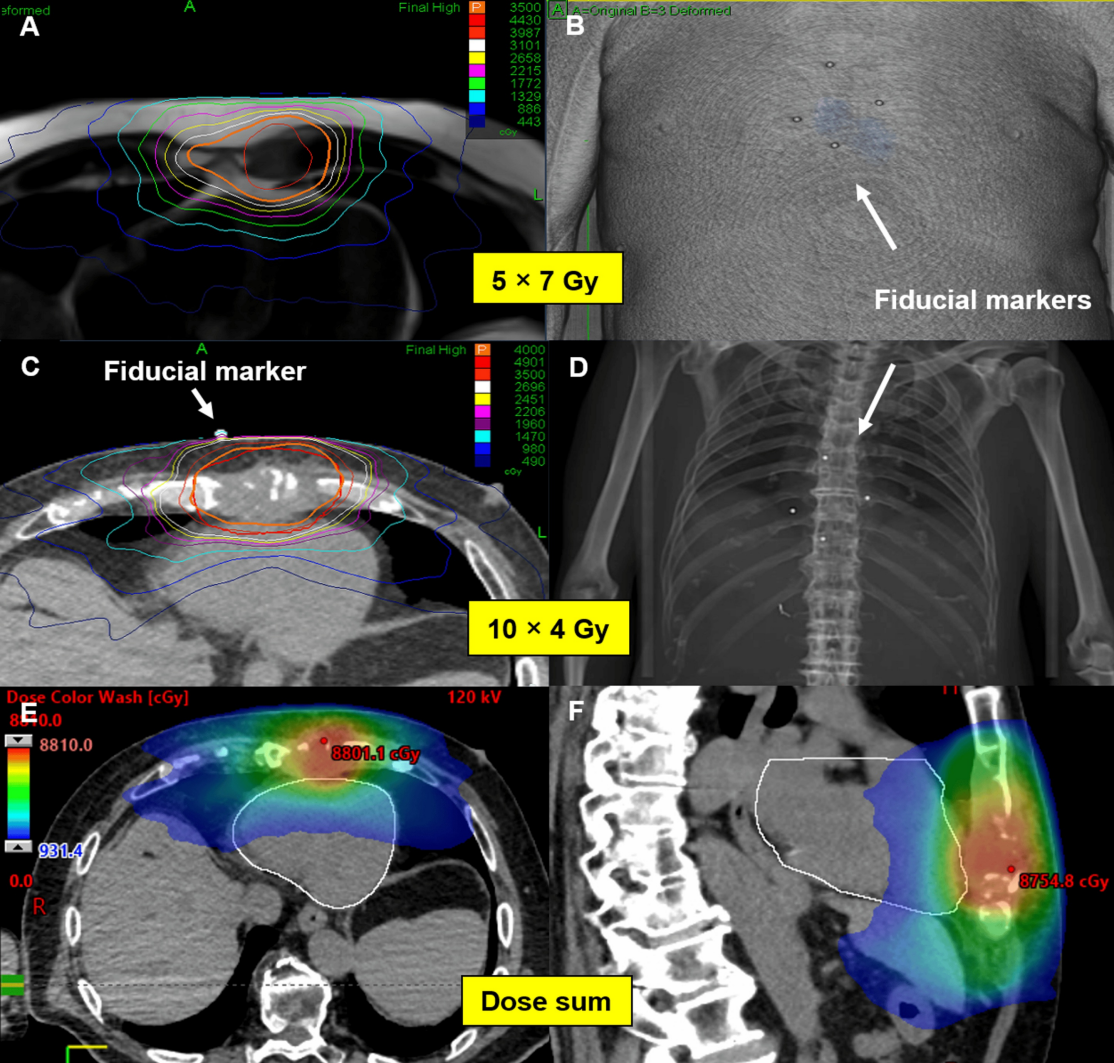
First SBRT with 5 × 7 Gy (A) and FMs placed in close proximity to the sternal lesion on the skin (B). Positioning of the FMs were marked with tattoos at planning CT to ensure consistent positioning of the FMs for each fraction. For the second SBRT (C) 10 × 4 Gy was delivered. Since the tattoos from the first treatment course were still visible, the FMs could be positioned at the exact same position for the second SBRT, as shown in the planning CT and DRR (C, D). The bottom two images (E, F) show a dose summation in color wash of both SBRTs. Notably, in this plan sum the mean and maximum heart doses were 11.3 Gy and 81 Gy, respectively, while 15 cc of the heart received 41.4 Gy, remaining within the D15cc dose constraint of 42 Gy in 15 fractions as recommended by Timmerman. SBRT = stereotactic body radiotherapy, FMs = fiducial markers, DRR = digitally reconstructed radiograph Timmerman [4]

A 77-year-old man with metastatic hepatocellular carcinoma (mHCC) was referred to our department for SBRT of a sternal metastasis. At the time of the initial diagnosis, the tumor was localized to liver segment III and was surgically resected. Two and a half years later, the disease recurred with metastases in the abdominal wall, adrenal gland, lungs, and sternum. Systemic therapy was initiated, but due to further tumor progression, treatment was switched to immunotherapy with nivolumab. This led to the regression of all metastatic sites. However, 18 months after starting immunotherapy, asymptomatic disease progression was observed exclusively in the sternal metastasis. Given the isolated recurrence, SBRT was administered to the sternal metastasis with a dose of 5 × 7 Gy using the CyberKnife® with skin surface FMs (Figure 1), while nivolumab was continued unchanged.

**Figure 2:**
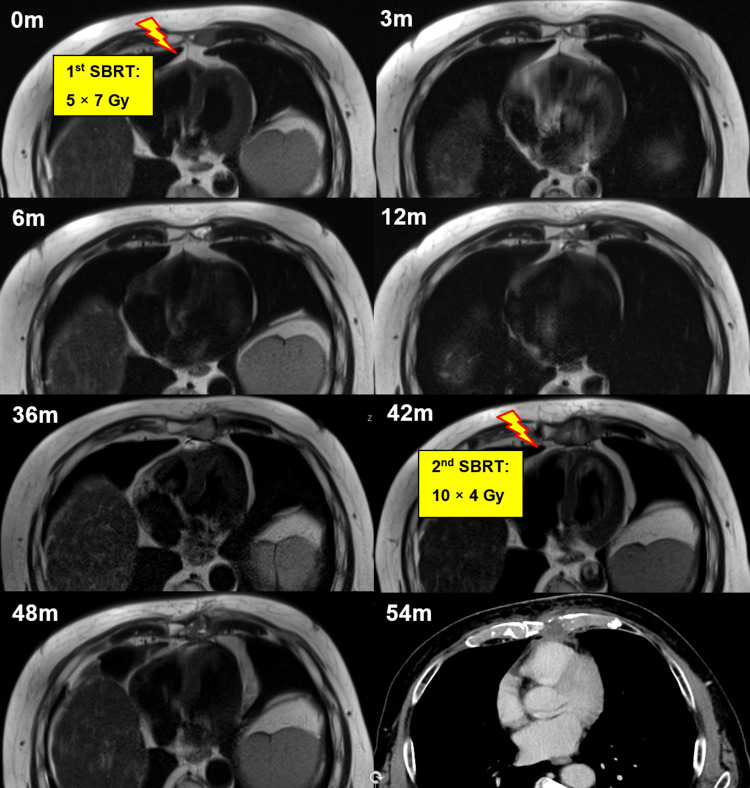
T2-weighted MR images showing the sternal metastasis over time. At baseline (0m) the first SBRT with 5 × 7 Gy was delivered using the CyberKnife (Accuray Inc., Sunnyvale, CA). Follow-up scans were performed at three, six, 12, and 36 months (3m, 6m, 12m, 36m). After local progression was confirmed at 42 months (42m), re-SBRT with 10 × 4 Gy was delivered with the CyberKnife. MRI and CT images confirmed that the lesion remained controlled at six and 12 months after re-SBRT (48m, 54m). The patient died 14 months after re-SBRT due to multifocal progression. SBRT = stereotactic body radiotherapy

Follow-up evaluations at three and six months post-treatment showed that the patient maintained good performance status and remained free of sternal pain or other symptoms. Radiological assessment following SBRT demonstrated significant post-treatment changes on magnetic resonance imaging (MRI). Specifically, a marked reduction in alterations in T2-weighted sequences, characterized by a mixed pattern of hyperintense and hypointense signals, suggested a favorable radiological response up to 36 months after SBRT (Figure 2). At 42 months post-treatment, an MRI confirmed the progression of the sternal metastasis. The disease was otherwise controlled, and a biopsy of a newly detected liver lesion showed no malignancy. Given the isolated progression in the sternum, the patient underwent re-irradiation with the CyberKnife® (10 × 4 Gy over two weeks, see Figures 1, 2) using the previously mentioned approach while nivolumab was continued. The fractionation scheme for re-irradiation was selected to ensure that, in the cumulative dose plan including the initial SBRT plan, heart dose constraints were respected in accordance with the thresholds defined by Timmerman, as previously described [4].

Before re-irradiation, the patient reported mild sternal pain of 3 out of 10 on the visual analogue scale (VAS). One month after re-irradiation, he was pain-free. At six months following re-irradiation (48 months after initial SBRT), the sternal metastasis remained controlled (Figure 2). However, one year after re-irradiation (54 months after the first SBRT), the patient developed multifocal progressive disease with newly diagnosed ascites, hepatic, osseous, and soft tissue metastases. Computed tomography (CT) imaging showed that the sternal lesion remained controlled, but due to his drastically declining general health and poor prognosis, nivolumab was discontinued, and best supportive care was initiated. The patient succumbed to the disease two months later, 14 months after the second SBRT to the sternal metastasis.

Case 2

**Figure 3:**
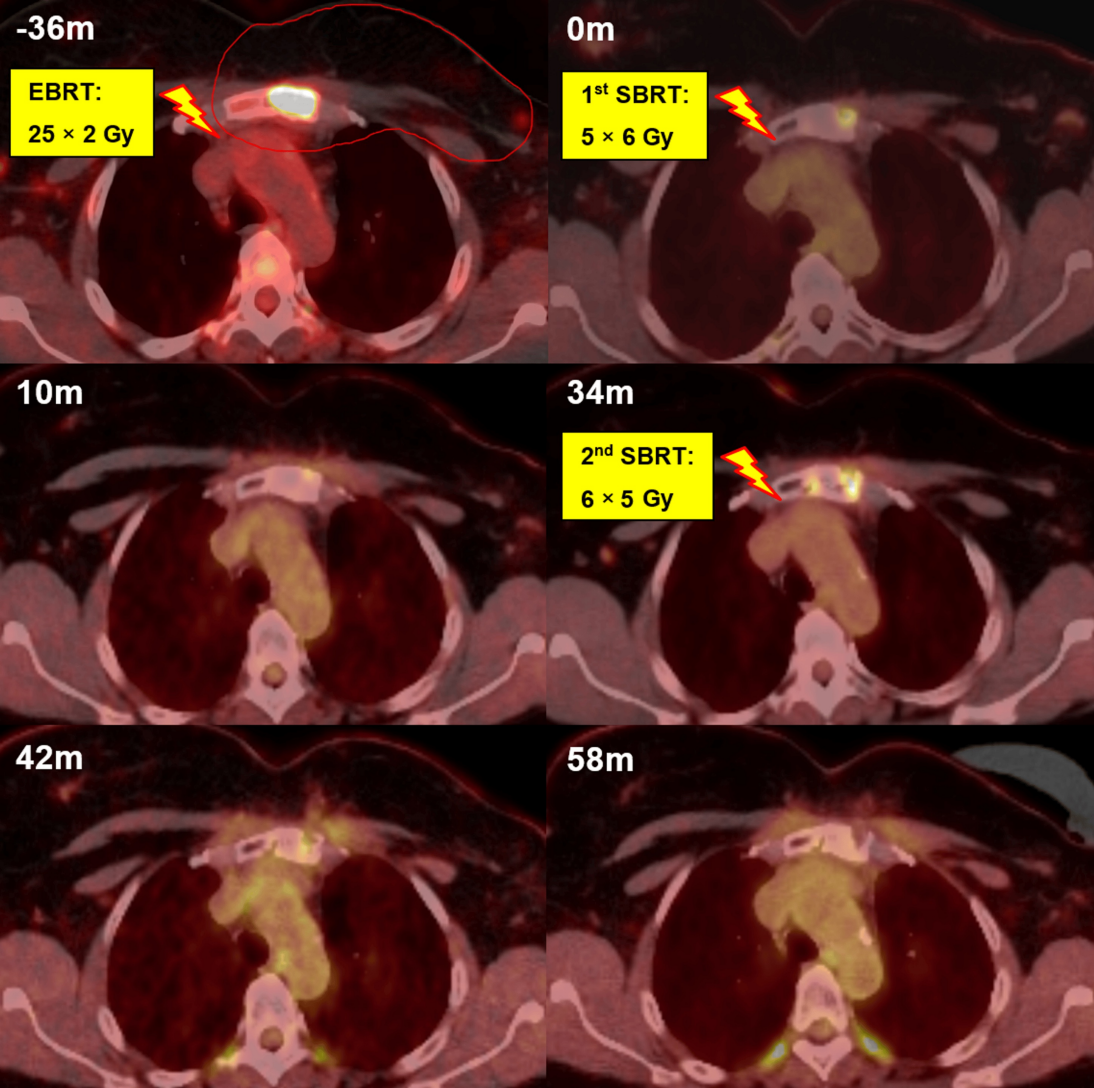
PET/CT images showing the sternal metastasis over time. The metastasis was included in thoracic wall irradiation following the initial recurrence three years before SBRT (-36m). At baseline (0m), the first SBRT with 5 × 6 Gy was delivered. Follow-up PET/CT scans were performed at 10 and 34 months (10m, 34m). After local progression was confirmed with biopsy at 34 months, a second SBRT with a dose of 6 × 5 Gy was administered (34m). PET/CT and CT images confirmed that the lesion remained controlled at eight and 24 months after re-SBRT (42m, 58m). The patient was still alive at the last follow-up, two years after re-SBRT. SBRT = stereotactic body radiotherapy

A 49-year-old woman with metastatic breast cancer and a solitary sternal metastasis was referred for SBRT evaluation. Her breast cancer had been initially diagnosed 15 years earlier and managed with mastectomy, chemotherapy, and hormonal therapy. Eleven years later, she developed a chest wall recurrence and a metastasis in the manubrium. Following a multidisciplinary discussion, the chest wall recurrence was treated with surgery, intraoperative radiotherapy (IORT), adjuvant EBRT, and continued hormonal therapy. The EBRT treatment plan also encompassed the region of the internal mammary artery and the sternal metastasis, delivering a total dose of 50 Gy in 25 fractions. The disease remained stable for three years. The patient subsequently developed diffuse sternal pain (VAS 5-6/10), and a positron emission tomography-computed tomography (PET/CT) scan revealed increased metabolic activity in the previously treated sternal lesion. A CT-guided biopsy confirmed relapse. Given the history of prior EBRT to the chest wall and sternum, SBRT with 5 × 6 Gy was delivered to the progressive sternal metastasis. Follow-up imaging demonstrated both metabolic and morphological response, characterized by the disappearance of tracer uptake in PET/CT and sclerotic changes (Figure 3).

The patient reported a partial reduction in pain (VAS 2-3) at follow-up visits. The disease was controlled with tamoxifen for three years following SBRT until the development of thoracic skin metastases and progression of the sternal metastasis prompted surgical resection of the skin metastases and an escalation of systemic therapy to letrozole and ribociclib. Due to the oligoprogressive nature of the disease, the patient was referred for re-irradiation of the sternal metastasis. Re-SBRT with 6 × 5 Gy was delivered to the sternal lesion using the same approach with the CyberKnife® and skin FMs, again ensuring that heart dose constraints were respected in the cumulative dose from both the initial and re-SBRT plans. The disease remained controlled for two years until the last follow-up, with the patient being symptom-free and employed.

**Figure 4:**
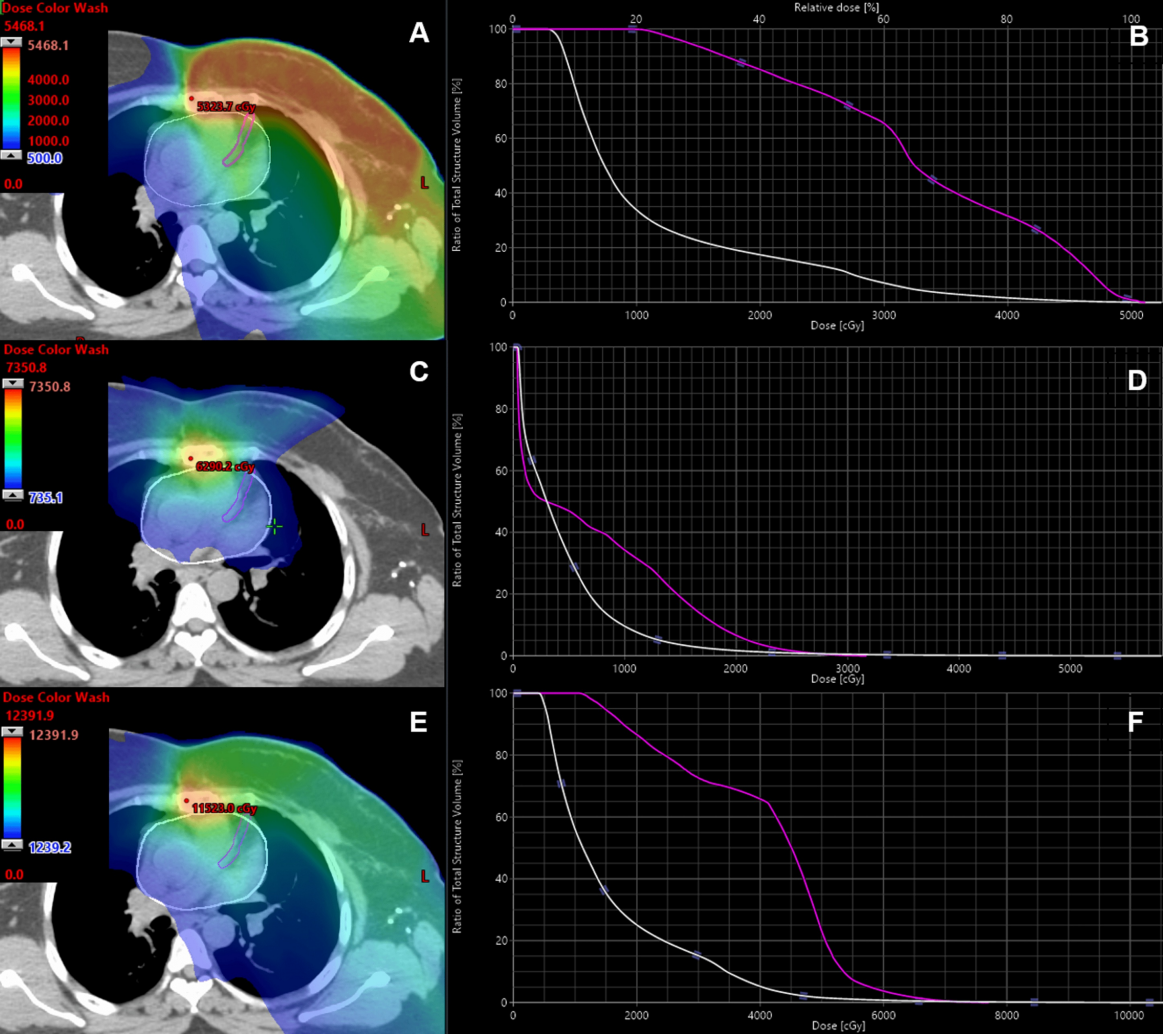
Dose distribution of the EBRT plan after diagnosis of the first relapse (A) and the dose-volume histogram (DVH) of the LAD (B, purple line) and heart (B, white line). Maximal and mean dose to the LAD was 55.2 Gy and 33.1 Gy, respectively. Mean heart dose was 11.4 Gy. Panel C shows the dose distribution resulting from summation of both SBRT plans, with the corresponding DVH for the LAD and heart shown in panel D. Here, the LAD received a maximum dose of 31.7 Gy and a mean dose of 7.1 Gy; the mean heart dose was 4.4 Gy while 15 cc of the heart received 16.1 Gy, remaining well within the D15cc dose constraint of 36 Gy in 10 fractions as recommended by Timmerman. The bottom images (E, F) show a dose summation of all SBRT and EBRT plans. In the plan sum, the maximum and mean doses to the LAD were 77.2 Gy and 40.3 Gy, respectively, with a 15.9 Gy mean heart dose. EBRT = external beam radiotherapy, SBRT = stereotactic body radiotherapy, DVH = dose-volume histogram, LAD = left anterior descending artery Timmerman [4]

Notably, seven years after thoracic EBRT and four years after the first SBRT to the manubrium, the patient required left anterior descending artery (LAD) stenting due to significant stenosis, corresponding to a grade 3 toxicity according to the Common Terminology Criteria for Adverse Events (CTCAE), version 5 [7]. Alongside multiple thoracic irradiations, the patient has additional cardiac risk factors, such as hypertension, hypercholesterolemia, and a family history of myocardial infarction. Dose-summation of all SBRT and EBRT treatment plans was performed to assess the dose delivered to the LAD. Combining all irradiations, the maximum and mean doses to the LAD were 77.2 Gy and 40.3 Gy, respectively. However, given the metastasis location in the manubrium, the primary LAD dose contributor was the initial EBRT, as shown in Figure 4.

## Discussion

The use of SBRT for non-spine metastases has increased substantially in recent years, but data on SBRT for sternal metastases remain scarce. At our institution, we standardized SBRT for sternal metastases using skin FMs with real-time motion synchronization. Since this is a completely non-invasive approach, it avoids complications associated with traditional invasive gold marker implantation used alongside Synchrony®, such as pain, hemorrhage, pneumothorax, pleural effusion, or FM migration [8,9]. In addition, patients can breathe freely, making this technique suitable for those who struggle with breath-hold during respiratory gating approaches. Furthermore, the CyberKnife Xsight® Spine Tracking System (Accuray Inc., Sunnyvale, CA), which relies on bony landmarks for real-time target tracking, was not designed for non-spinal metastases and is, therefore, not applicable for treating sternal lesions.

Here, we present two re-irradiation cases of sternal metastases treated twice with SBRT using skin FMs with real-time motion synchronization. Advances in anti-cancer therapy have led to extended survival even in metastatic patients. Therefore, it is crucial to treat these patients as precisely as possible while minimizing the dose to organs at risk, aiming to reduce both acute and late toxicity. In both re-irradiation cases presented, we were able to maintain in the plan sum of both SBRTs relatively low cumulative mean heart doses of 11.3 Gy and 4.4 Gy, respectively. Additionally, the heart volume constraint D15cc, as proposed by Timmerman, was respected in the plan sum in both cases [4]. Both patients tolerated re-SBRT well and achieved complete pain relief. No late toxicity was observed in the first case; however, a significant LAD stenosis requiring intervention was diagnosed in the patient with metastatic breast cancer (mBC). The cumulative radiation dose to the LAD from three irradiations may have contributed to this late grade 3 toxicity (CTCAE, version 5) [5]. However, the patient also has significant additional cardiac risk factors, which makes it difficult to determine the exact cause of the LAD stenosis. Extensive data from breast cancer studies show that patients with left-sided breast cancer have a higher risk of cardiac mortality, and irradiation dose to cardiac structures should be kept as low as possible (mean heart dose <5 Gy and mean LAD dose <10 Gy) [10,11]. Darby et al. found that each additional 1 Gy mean heart dose increases myocardial infarction risk by 7.4%, while McKenzie et al. reported an association between LAD V15 Gy ≥10% and increased all-cause mortality [12,13]. These findings highlight the importance of precise irradiation and sparing of cardiac tissue.

Remarkably, in both patients, durable local control of the sternal metastasis was achieved after re-SBRT. This is particularly significant because relapsed and pre-irradiated tumors are considered more radioresistant and aggressive. Compared to traditional free-breathing irradiation, real-time motion synchronization with CyberKnife® allows for smaller irradiation volumes and reduces dose exposure to normal tissue while delivering ablative doses to the tumor. Currently, we are optimizing SBRT for sternal metastases with a focus on reducing the mean heart dose and respecting the SBRT constraints proposed by Timmerman [4]. We are evaluating further optimization of the SBRT plan for the LAD, which may help further reduce cardiovascular toxicity.

## Conclusions

We report two re-irradiation cases of sternal metastases treated twice with SBRT using skin surface FMs and real-time motion synchronization. Both patients tolerated the treatment well, achieving complete pain relief and durable local control. The use of skin FMs to mitigate respiratory motion is a non-invasive, simple, and effective approach. We are currently investigating SBRT plan optimization with an additional focus on LAD sparing to further reduce cardiovascular toxicity.
